# L-lysine moderates thermal aggregation of coconut proteins induced by thermal treatment

**DOI:** 10.1038/s41598-023-38758-7

**Published:** 2023-08-16

**Authors:** Liqiang Wang, Youbang Zhang, Run Li, Dong Xiang

**Affiliations:** 1 School of Food Science and Engineering, Hainan University, No. 58 Renmin Avenue, Haikou, 570228 China; 2 Key Laboratory of Food Nutrition and Functional Food of Hainan Province, No. 58 Renmin Avenue, Haikou, 570228 China

**Keywords:** Analytical chemistry, Biochemistry

## Abstract

This work attempts to investigate the inhibitory effect of L-lysine (Lys) on the thermal aggregation of coconut protein (CP). The results showed that under neutral conditions (pH = 7), temperature reduced the solubility and enhanced the thermally induced gel formation of CP. In addition, Lys reduced the fluorescence properties, particle size and increased the turbidity of CP, which had an inhibitory effect on heat induced gels. The results indicate that Lys plays an important role in inhibiting protein thermal aggregation by interacting with CP to create steric hindrance and increase protein electrostatic repulsion.

## Introduction

Coconut (*Cocos nucifera* L.) is a high-quality perennial plant with high ecology and economic value^1^. Coconut plays a vital role in the economic development and food production of Southeast Asian countries, such as Thailand, Laos, Myanmar, the Philippines, and South Asian country India^2,3^. Mature coconut contains fat, protein, polysaccharide, and other trace elements^4^. Currently, coconut oil, coconut milk, and solid coconut endosperm products have widely used in the food industry^5,6^. Coconut protein (CP) had obtained from the solid endosperm in the oil-seed coconut. CP has biological effects such as reducing the absorption of cholesterol and the accumulation of fat^4,7,8^.

CP and some oilseed proteins are of broad interest due to their poor solubility^9,10^. Insoluble proteins are heated to produce thermal aggregates^11^. Autoclaving is an essential step in the processing of coconut products^12,13^. Undiluted coconut milk sterilized directly at autoclave becomes unstable and appears as a non-flowing solid^13^. This immobile solid structure attributes to the denaturing and unfolding of proteins after autoclaving. Interactions between adjacent proteins occur, forming a three-dimensional gel network that encases other substances such as water and fat^14,15^. It has a negative impact on the production and sale of products. Therefore, finding a substance to inhibit the inability of CP in coconut pulp products to form a three-dimensional mesh structure during autoclaving has high significance in broadening the applications of coconut pulp in the food industry.

Recently, it was reported that amino acids (lysine, histidine, arginine) improve the solubility, texture, and interfacial tension of meat proteins^16,17^, increase the stability of soy protein emulsions^18^, and improve the quality of frozen white shrimp^19^. Therefore, the extensive use of amino acids in the food industry has attracted the interest of researchers^18,20,21^. In essence, protein solubility is affected by the interaction between proteins and the interaction between proteins and water. On the one hand, analysis of fluorescence properties and particle size distribution showed that histidine inhibits the aggregation of myosin mass and increases its solubility^22^. On the other hand, additives that modify the interaction between proteins and water explain themselves mainly based on three different approaches: interactions between additives and proteins (preferential interactions), interactions between additives and amino acids (amino acid solubility), and interactions between additives and water (e.g., ionic hydration or surface tension interactions)^23–25^. Such interactions can increase the steric hindrance between proteins and reduce the chance of protein–protein interactions^26^. It is not clear whether the effect of Lys on CP solubility is similar. Therefore, this study sought to explore the effect of different levels of Lys (0–0.3%, w/v) on low concentration (1%, w/v) CP solutions using solubility, turbidity, fluorescence properties, and particle size; further rheological tests and scanning electron microscopy (SEM) were used to investigate the effect of different levels of Lys on the least gelation concentrations(LGC) (5% and 9%, w /v) CP to improve the availability of coconut pulp in the food industry and to generate economic benefits.

## Results and discussion

### Effect of heat treatment on CP solution

**Figure 1:**
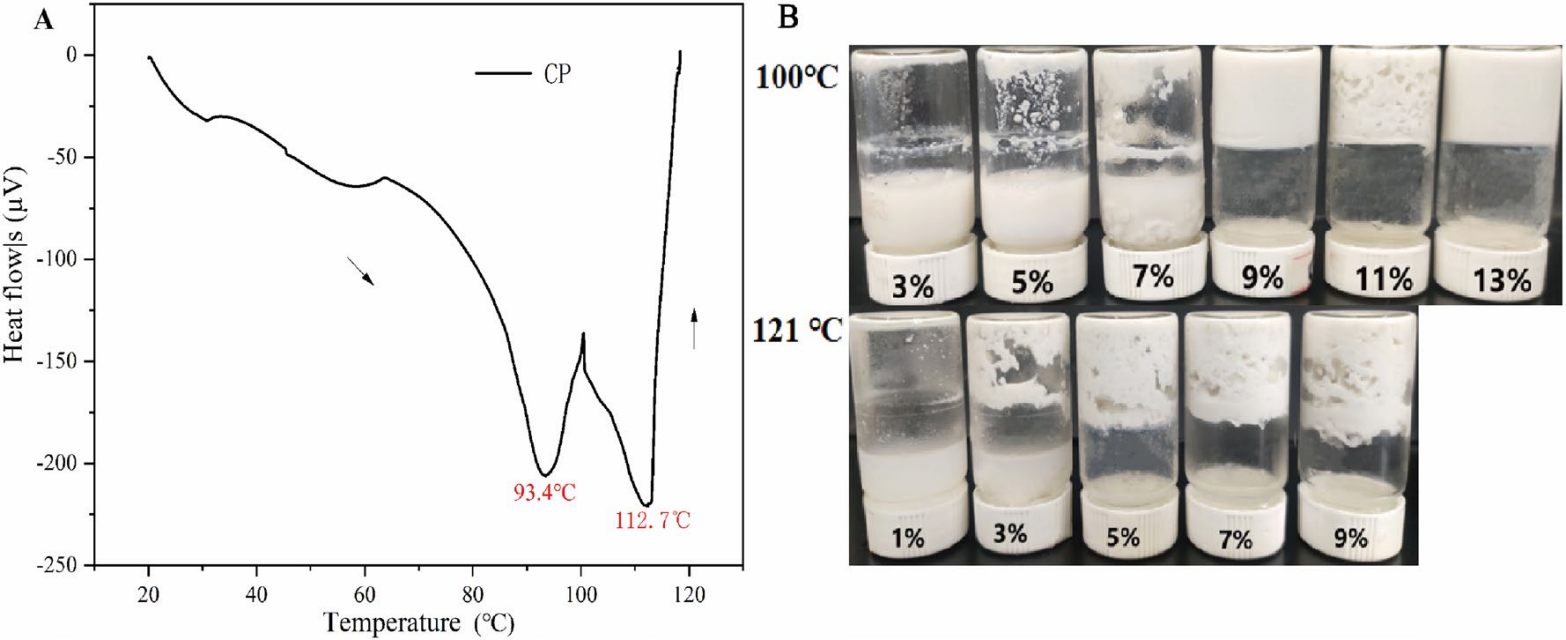
Thermal analysis of CP. (**A**) DSC profiles of CP; (**B**) Effect of 20 min heat treatment at different temperatures (100 °C, 121 °C) on CP solutions.

The thermal performance of the CP was evaluated under neutral conditions(pH = 7) by differential scanning calorimetry (DSC)^27^. The thermal analysis curve shows two broad heat absorption peaks (Fig. 1A). It was probably due to the thermal denaturation of the 7S and/or 11S globulin fractions^27^. The T_d_ of CP was around 93.5 °C and 112.7 °C, similar to the results of the Kwon^28^. It shows that the protein molecules continuously absorb energy during the heat treatment^29^. This energy breaks the internal covalent and non-covalent bonds, denaturing and unfolding the protein, thus altering the tertiary structure and rendering the protein structure disordered^30^.

LGC is an important indicator of thermally induced gel formation of proteins^31^. When the concentration of protein in the solution exceeds a critical concentration, thermally induced gel with a certain shape is formed upon heating^31^. Figure 1B shows LGC of the CP solution after heat treatment at different temperatures. After heat treatment at 100 °C, at low concentrations of CP solution, the gel forms a looser structure, causing a small portion of the protein solution or gel mass to slide down to the mouth of the bottle in a semi-solid state. When the concentration of CP solution was above 9%, the protein formed a heat-induced gel and the gel did not slide down to the bottom of the reagent bottle after inversion. Heat treatment of the CP solution at 121 °C at a concentration of 5% is sufficient to form a heat-induced gel. These results suggest that the LGC is closely related to protein concentration and temperature^32^. Therefore, subsequent verification of the ability of Lys to moderate the thermal aggregation of CP using thermally induced gels was also based on LGC.

### Lys-CP mixture properties

**Figure 2:**
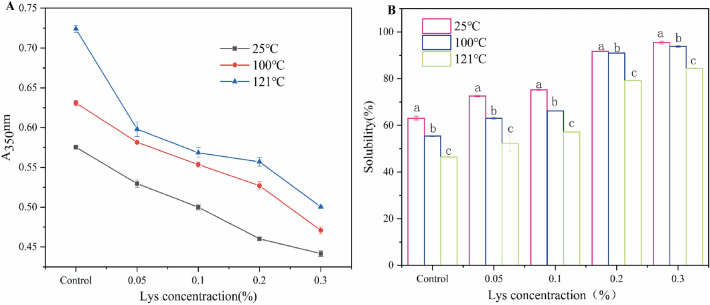
Properties of Lys-CP mixture after different heat treatments (25 °C, 100 °C, and 121 °C); (**A**) Turbidity. (**B**) Solubility.

Changes in turbidity reflect the degree of protein aggregation, while solubility reflect the number of insoluble aggregates in the protein solution^33^. Turbidity and solubility were measured for Lys-CP mixtures with different levels of heat treatment (25 °C, 100 °C, and 121 °C). After heat treatment, the turbidity of the CP solution increased from 0.57 to 0.72; the solubility decreased from 63.09 to 46.39% (Fig. 2). It is possible that the heat treatment can denature and aggregate the proteins, producing aggregates^34^. The presence of aggregates reduced the solubility and increased the turbidity of the CP solution^35^. When Lys was added and heat treated, turbidity decreases from 0.57 to 0.44 at 25 °C; from 0.63 to 0.47 at 100 °C; and from 0.72 to 0.50 at 121 °C (Fig. 2A). The solubility increased from 63.1 to 95.4% at 25 °C; from 55.4 to 93.8% at 100 °C; and from 46.4 to 84.4% at 121 °C (Fig. 2B). The results showed that the addition of Lys could improve the solubility and reduce the turbidity of the CP solution after heat treatment. Similarly, Lys has been reported to increase the solubility of proteins. A few authors have hypothesized that Lys can increase the electrostatic repulsion of proteins through interactions with them (e.g., electrostatic interactions)^36^. Due to spatial site resistance, protein interactions are reduced, and solubility is increased, which can slow down protein thermal aggregation^19,37^. Therefore, Lys may increase CP solubility and alleviate CP thermal aggregation.

Subsequently the effect of heat treatment temperature and Lys content on low concentration CP solutions was further investigated by changes in particle size distribution and protein tertiary structure.

### Effect of Lys on the fluorescence characteristics of CP solutions

**Figure 3:**
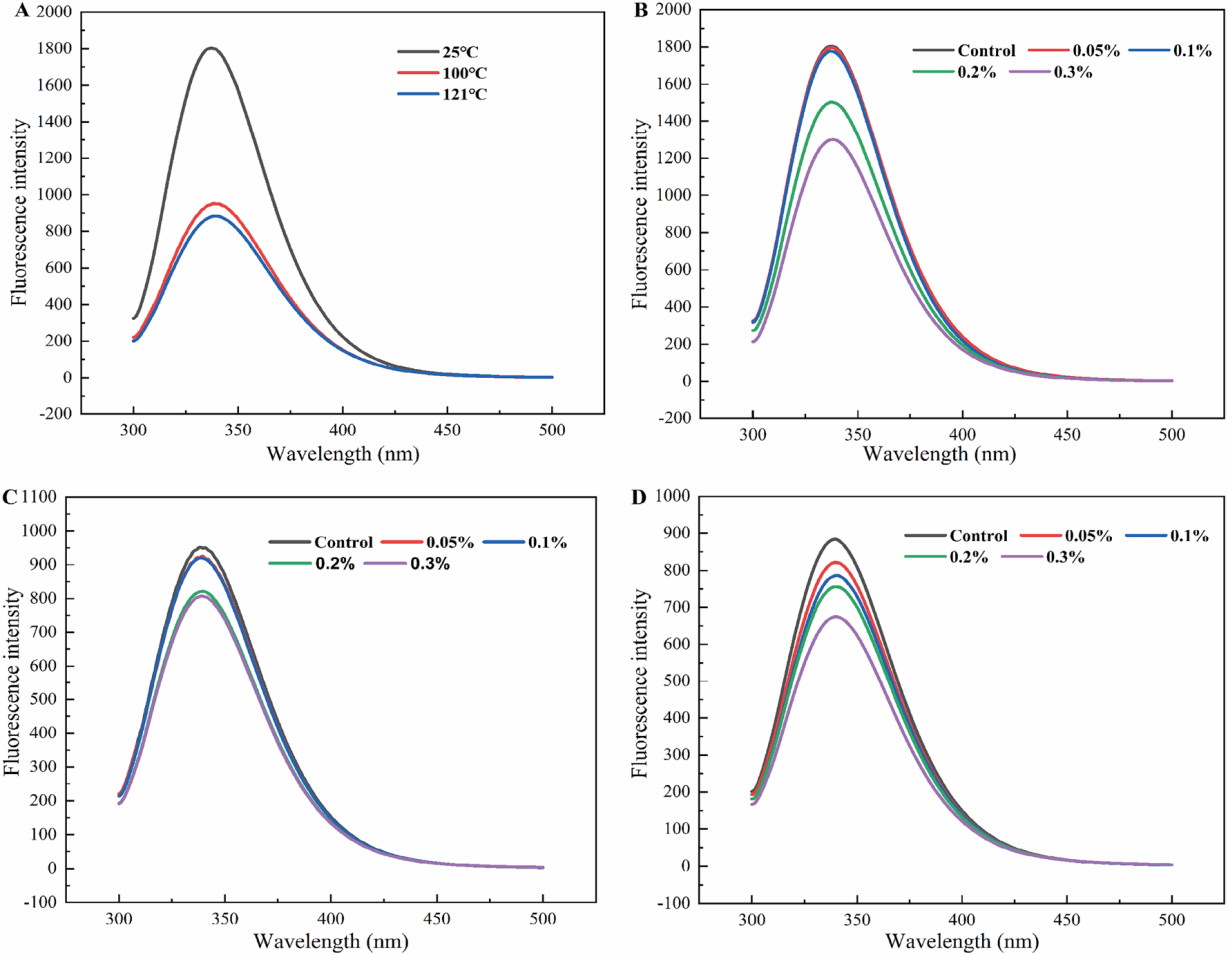
Change in fluorescence spectroscopy scanning of Lys-CP solution. (**A**) FI of heat-treated CP solutions at different temperatures; (**B**–**D**) FI of heat-treated Lys-CP mixture at different temperatures (25 °C, 100 °C, 121 °C).

Tryptophan (Try) is typically used to measure changes in the tertiary structure of proteins due to its sensitivity to the hydrophilic environment^38^. It is widely accepted that folded proteins exhibit high fluorescence intensity (FI)^39^. When proteins undergo denaturation unfolding, try was exposed to a polar microenvironment (λ_max_ > 330 nm), resulted in a decrease in FI^22^. As shown in Fig. 3A, the FI of CP decreased after heat treatment. Attributed to the heat treatment caused the protein denaturation to unfold, and the fluorescence quenching occurred by exposing the internal tryptophan residues^40^. Heat treatment after the addition of Lys to the CP solution revealed a decrease in FI and red shift (Fig. 3B–D). It is possible that the addition of Lys caused the unfolding of the CP tertiary structure^41,42^. Similarly, Li et al.^42^ induced unfolding of myosin by adding Lys, leading to a decrease in FI and the appearance of red shift. Probably, this is due to the cation-π interaction of Lys with tryptophan residues, which changes the microenvironment around the tryptophan residues^37^. After heat treatment, the decrease in FI was more pronounced. Perhaps the interaction of Lys with the tryptophan residues unfolded by protein denaturation was caused. Our results suggest that the presence of Lys can induce changes in the tertiary structure of CP and a more polar environment for tryptophan residues.

### Effect of Lys on the particle size of CP solutions

**Figure 4:**
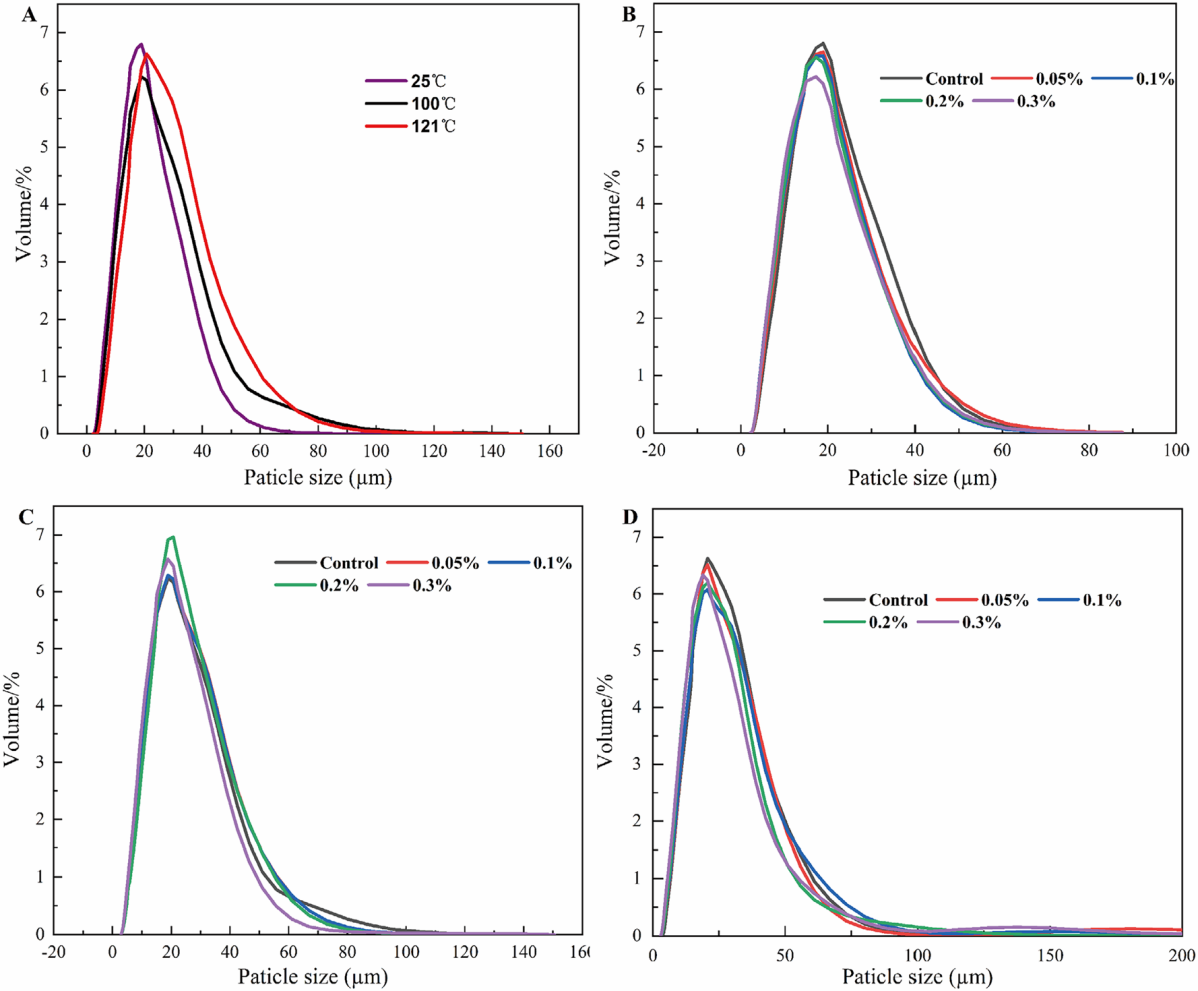
Effect of Lys on the particle size distribution of CP particles. (**A**) Particle size distribution of CP solutions under heat treatment at different temperatures; (**B**–**D**) Particle size distribution of Lys-CP solutions under heat treatment at different temperatures (25 °C, 100 °C, 121 °C).

The particle size distribution maps can reflect the degree of protein aggregation and characterize the spatial structural changes of protein^43^. Figure 4A shows the particle size distribution of the CP solution after heat treatment. We can find that the particle size distribution of the protein solution was significantly changed (*P* < 0.05) and the number of aggregates of macromolecules increased with the increase of heat treatment temperature. Temperature is proportional to the size of the thermal aggregates. Thus, high temperature treatment can cause a series of covalent and non-covalent interactions between adjacent proteins, which leads to the aggregation of proteins and the formation of aggregates^44^. Figure 4B–D shows the effect of Lys content on the particle size of CP solutions at different temperature conditions. Under neutral conditions (pH = 7), the average particle size of the protein solution decreased with increasing Lys content, indicating that the aggregation of the Lys-CP mixture was inhibited after heat treatment. On the one hand, Lys undergoes cation-π interactions with tryptophan residues, changes the protein surroundings to be more polar^37^. On the other hand, weak interaction of Lys with proteins leads to a reduction in protein–protein interactions and a spatial site blocking effect^45^. This spatial site blocking effect provides favorable conditions for inhibiting the formation of thermal aggregates^36,46,47^. When the formation of aggregates is improved, the particle size of CP thermal aggregates decreases, which is consistent with previous results on the effect of Lys on the turbidity and solubility of CP thermal aggregates.

Further to demonstrate that Lys can increase the solubility of CP and alleviate the formation of thermal aggregates. We added Lys to 5% (w/v) and 9% (w/v) CP solutions for different temperature heat treatments. It was verified whether the thermally induced gel could also be formed.

### Rheological properties

**Figure 5:**
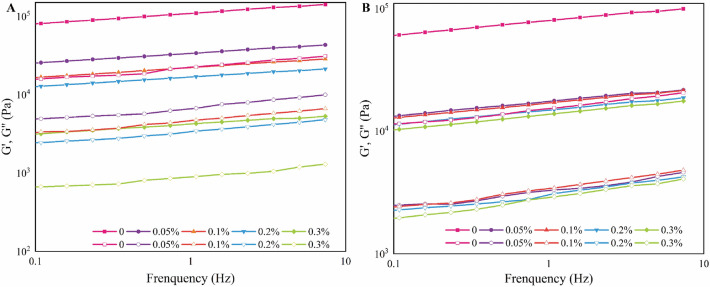
Rheological properties of Lys-CP solutions at minimum gel concentration (5% and 9%). (**A**) 100 °C for 20 min; (**B**)121 °C for 20 min.

The energy storage and loss modulus of Lys-CP thermally induced gels versus angular frequency (Fig. 5). When the angular frequency increased, the G′ and G″ of the gel increased, showing a strong frequency dependence. Throughout the measurement range, the higher the concentration of Lys added, the smaller the G′ and G″ values, which was attributed to the interaction of Lys with the proteins (caption-π interactions), reducing the interactions between the proteins^37^. With fewer protein interactions, the size and number of thermal protein aggregates decreased, and ultimately the chance of thermally induced gel generation is suppressed^37^. Guo et al.^16^ showed that the protein stabilizer Lys significantly increased the solubility of proteins, thereby altering the texture and water retention of meat products. In addition, Lys raises the pH of the protein solution^48^. Lys is an alkaline amino acid that regulates the pH to keep proteins away from pI^19^. As the pH moved away from pI, the negative charge in protein solutions increases, repulsion between proteins increases, sites for binding water are enhanced, and the ability to form protein gels is diminished^49^. Thus, the strongly hydrophilic Lys enhances protein-water interactions. However, Li et al.^24^ reported that the addition of Lys and Arg, with pH at myosin pI also increased their solubility. As a result, the increase in protein solubility may be the result of a combined effect.

### SEM

**Figure 6:**
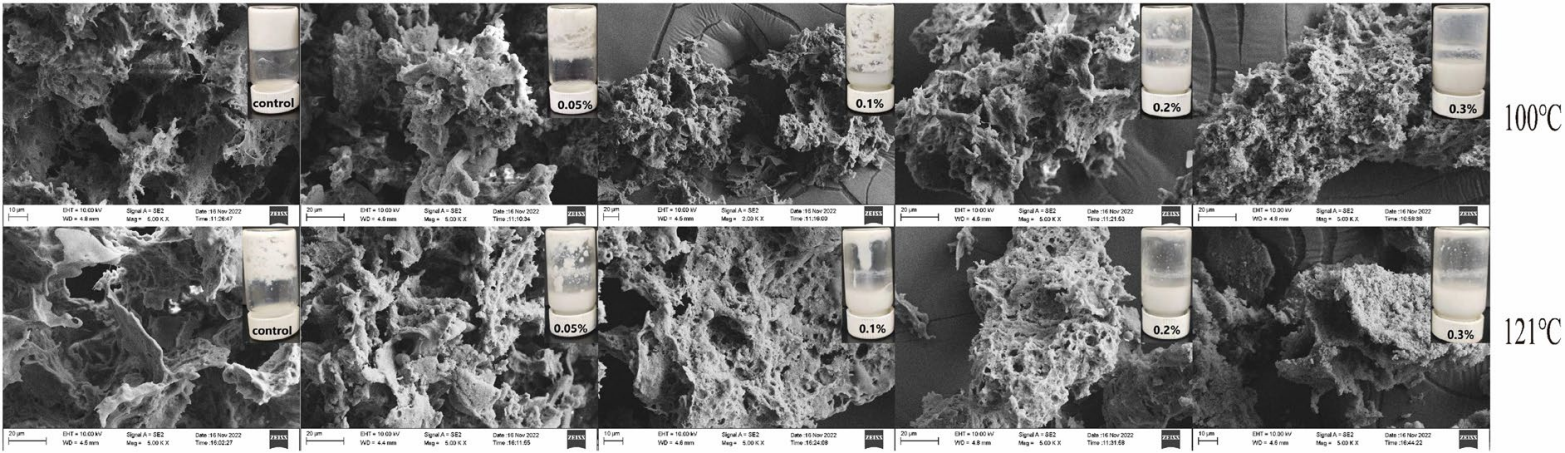
SEM images of CP gels at different concentrations of Lys (0–0.3%, w/v).

At different temperature conditions (100 °C, 121 °C), the formation of CP thermally induced gels diminished with increasing amounts of Lys added. The CP solution was unable to form thermally induced gels at Lys content of 0.2% (w/v). The SEM images also confirmed this view (Fig. 6). CP thermally induced gels show rough surfaces, large cavities and uneven porosity. The three-dimensional surface of the thermally induced protein gels formed a flatter and more uniform structure with smaller pores after the addition of Lys^50^. It was attributed to the fact that the addition of Lys increased the solubility of the proteins (Fig. 2), altered the tertiary structure of the protein (Fig. 3), reduced the particle size of the protein (Fig. 4), decreased the rheological properties of the protein (Fig. 5), and induced the cross-sectional area of the protein to exhibit a smaller cavity distribution and smoother planes. These results further elucidate that Lys increases CP solubility, alleviates the generation of thermal aggregates and inhibits the formation of thermally induced gels. We followed up by exploring the mechanism of action of Lys inhibition of thermal aggregation by the formation of heat-induced gels from the main proteins of the different CP fractions. Subsequently, we explored the mechanism of action of Lys inhibition of thermal aggregation by the formation of thermally induced gels from different fractions of CP proteins.

### Exploring mechanisms

Natural proteins of plant and animal origin are thermally processed to produce insoluble thermal aggregates with reduced solubility during thermal processing. It is known as protein denaturation in food processing. When proteins undergo heat treatment conditions, the higher structure undergoes denaturation and unfolding, and the tight and ordered structure becomes a disordered structure peptide chain, which causes a loss of biological activity^51^. The physicochemical properties of denatured proteins are then changed, such as loose the peptide chain, reduced solubility, and precipitation^52^. When the advanced structure of a protein changes, the surface structure of the molecule changes, the hydrophilic groups are relatively reduced, and the hydrophobic groups hidden inside the molecule are exposed on the surface, making the protein particles insoluble with water. It is easy to cause molecules to collide and entangle with each other, and the phenomenon of dissociation and aggregation occurs in the thermal aggregation of proteins^53^.

**Figure 7:**
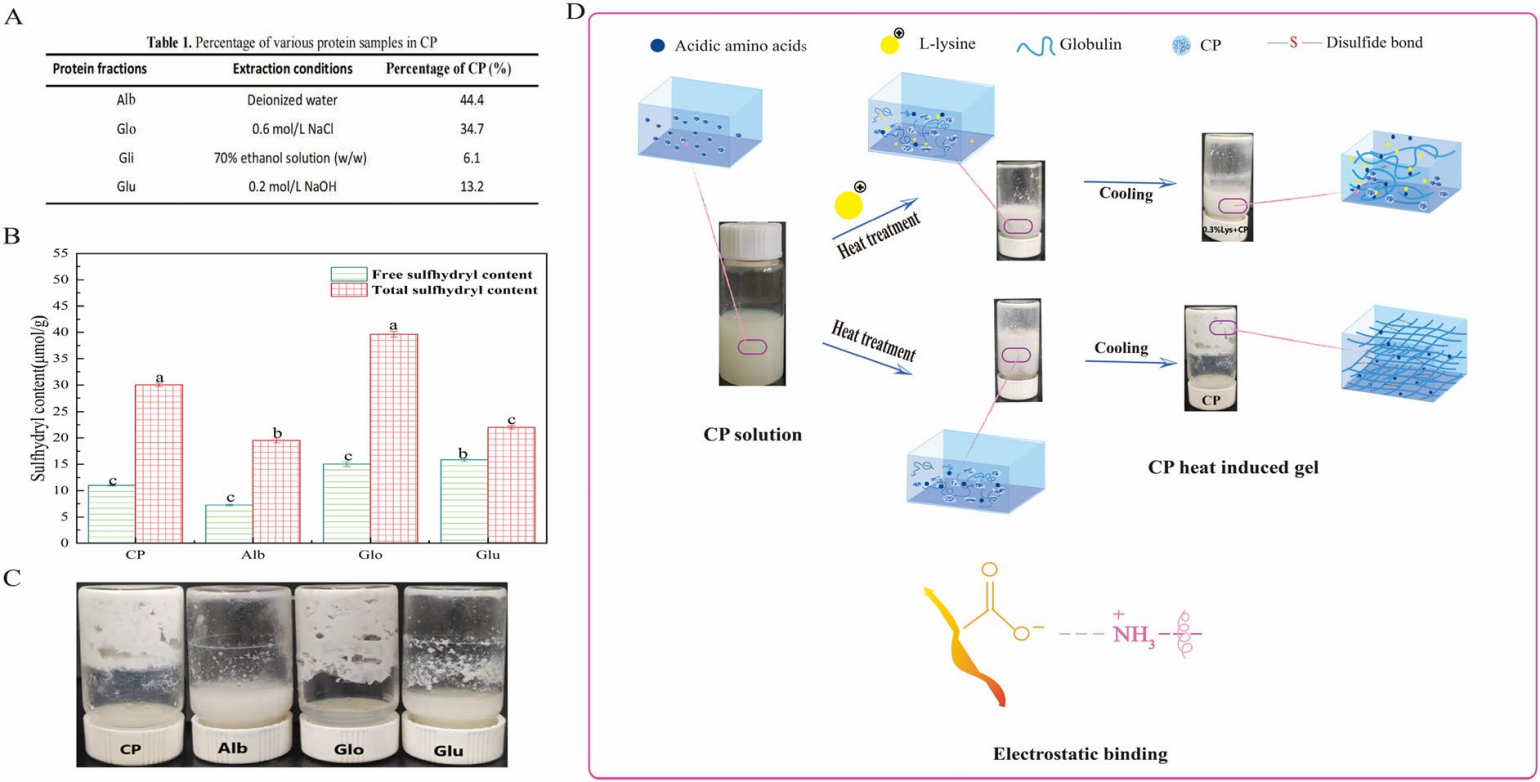
Exploration of the mechanism of Lys on CP heat aggregation. (**A**) Protein content of different fractions of CP. (**B**) Free sulfhydryl and total sulfhydryl content of different fractions of CP. (**C**) Apparent diagram of thermally induced gel formation in different fractions of CP at 121 °C for 20 min. (**D**) Diagram of the mechanism of CP thermal aggregation inhibition by Lys.

Several studies have shown that Globulin (Glo) are closely associated with the process of thermal denaturation of proteins, the primary mechanism of which is the formation of disulfide bond (S–S) from free sulfhydryl (–SH) groups in globulins through the oxidation of sulfhydryl groups, resulting in the formation of stable aggregates of proteins ^27,46,54^. We have further graded the CP and determined the content of different fractions, as shown in Fig. 7A. As shown in Fig. 7B–C, Glo has the highest total sulfhydryl content, followed by CP, Glutelin (Glu) and Albumin (Alb), indicating that the Glo contain more sulfhydryl groups within the molecule compared to other coconut fraction proteins, which is a prerequisite for thermal aggregation of CP to occur.

Glo contain not only more disulfide bond (S–S) but also more acidic amino acids (Asp and Glu, both containing γ-COOH)^55–57^. Some studies have reported that alkaline amino acids (lysine, arginine, histidine) to improve protein solubility, slow heat induced aggregation, and improve gel structure^16,17,25,42,58^. Ma et al.^59^ added L-Arg, L-Lys to soybean isolate to increase the solubility, emulsifying activity, and emulsion stability of soy protein. The mechanism of this action may be that after high temperature heat treatment, the acidic amino acid (Asp and Glu) residues within the Glo are exposed to the protein surface and surroundings, providing conditions for the interaction of Lys with Glo.

We hypothesize that the mechanism of action of Lys are shown in Fig. 7D. The possible mechanisms of action that exist are as follows:

Firstly, Lys is an alkaline amino acid which raises the pH of the protein environment. The increase in pH raises the negative charge on the protein surface, resulting in greater electrostatic repulsion between proteins. As the electrostatic repulsion increases, the monomeric structure of the protein also increases, lead to a reduction in protein interactions and a reduction in the binding sites for protein interactions, result in a reduction in the conversion of the (–SH) group into a disulfide bond (S–S)^60,61^.

Secondly, Lys contains charged groups such as ε-NH_2_, –COOH, –NH_2_, which readily interact with water and are highly hydrophilic. The positively charged Lys interacts with negatively charged amino acid residues on the protein surface to form complexes. The formation of complex leads to an increase in the hydrophilicity of the protein, which results in a weakening of the conversion of the hydrophilic group (e.g., –SH)) into a disulfide bond (S–S)^17^.

The experimental results of this study validate that Lys can alleviate thermal aggregation of CP induced by heat treatment.

## Methods

### Materials

Coconut was purchased from the aquatic wharf market in Haikou, Hainan, China. The collection of plant material and the experimental study of these plants comply with the national guidelines of China; Lys was purchased from Shanghai Yuanye Biotechnology Co., Ltd. (Shanghai, China). All solutions were prepared to use deionized water. All other chemicals are of analytical grade.

### Extraction of CP

CP: CP was extracted from coconut endosperm by isoelectric point precipitation. Solid coconut endosperm was removed from the coconut, cut into small pieces, mixed with phosphate buffer (PBS, pH = 8), and stirred. The mixture obtained was stirred continuously at room temperature for 6 h and filtered through 120 mesh gauze. CP mixture was centrifuged (6000 × g, 10 min) (CR22N, Hitachi, Japan), and the supernatant extracted. The supernatant was adjusted to pH = 4.5 with 1 mol/L hydrochloric acid solution, then left for 2 h to precipitate the protein. Proteins were precipitated by centrifugation at 10,000 × g for 10 min to extract the protein precipitate. Protein precipitates were dialyzed in dialysis bags (3800 Da retention capacity) for 48 h and then freeze-dried. The dry protein powder was defatted in hexane for 48 h to obtain CP. The total protein content of CP was determined by Kjeldahl nitrogen determination and using the protein conversion factor (N × 6.25) for all samples^62^. Further graded extraction of coconut protein (CP) was carried out by the method of Deng et al.^63^ to obtain albumin (Alb), globulin (Glo), gliadin(Gli) and glutelin (Glu). Protein content measured in solid endosperm was approximately 3.1 ± 0.21%, similar to the results of Kwon et al.^64^.

### Preparation of protein samples

A quantity of lyophilized CP powder was dissolved in deionized water and stirred magnetically at room temperature until all the protein had dissolved to make a protein solution with a mass concentration of 20% (w/v) and adjusted to pH = 7. A portion of the protein solution was diluted into different concentrations (3–13%) of CP solution, packed into sample bottles and subjected to different temperatures (100 °C, 121 °C) heat treatment for 20 min. The heat treatment CP dispersion was removed and placed in ice water, then stored in a refrigerator at 4 °C for 12 h before being removed. The fluidity of the protein samples was observed by inverting the vials. CP diluted into a low concentration (1%) and a high concentration protein suspension (5% and 9%). Various levels of Lys (0–0.3%) are added to the protein suspension to form a Lys-CP mixture and adjusted to pH = 7. The mixture was loaded into sample bottles and heat treatment for 20 min. Heat treatment samples were placed in a water bath in ice water and refrigerated at 4 °C for 12 h before removal. Completed samples were prepared and used for various studies. All samples in triplicate were processed. All samples were processed in triplicate.

### DSC thermal analysis

The protein samples were dissolved in deionized water to form a mixture, sodium azide (0.02%) was added as a preservative and mixed and transferred to a crucible. Samples evolved at 4 °C for 12 h and then the CP samples were measured by DSC (SETARAM, DSC131EVO, France) to obtain a thermal analysis profile. Sample conditions were as follows: A standard heating thermostat procedure consisting of heating the crucible containing the sample with the sample crucible (control) from 20 to 120 °C at a rate of 10 °C/min followed by a constant temperature at 120 °C for 20 min was set. Obtained images were analyzed using Calisto thermal analysis software. All samples were processed in triplicate.

### Effect of Lys on low concentration CP solutions

#### Determination of turbidity and solubility

Prepared Lys-CP mixture at 2.5 mg/L diluted with distilled water, and the turbidity of the solution at 350 nm was determined to use an enzyme marker (Biotek Instruments, Biotek, USA) and expressed as A350 nm. All samples were treated in triplicate.

Solubility (%) of the Lys-CP mixture as modified from Taşkın et al.^65^. Allow 10 mL of the prepared Lys-CP mixture to be centrifuged at 5000 × g for 10 min in a centrifuge (CR22N, Hitachi, Japan) and extract the supernatant. To 1 mL of supernatant, add 5 mL of Bradford's dye solution. After 10 min the absorbance of the protein was measured at 595 nm using an enzyme marker (Biotek Instruments, Biotek, USA). The soluble protein content (mg/mL) calculated using the standard curve for bovine serum albumin (BSA). Solubility (%) calculated as the percentage of protein concentration after centrifugation to the initial total protein concentration before centrifugation. All samples treated in triplicate.

#### Fluorescence spectroscopy scanning

The fluorescence spectroscopy scanning of the Lys-CP mixture was measured using an F-7000 fluorescence spectrophotometer (Hitachi Limited, Japan) at room temperature (25 °C). The measurement parameters were excitation wavelength 280 nm, emission wavelength 300–500 nm, excitation and emission wavelength slits 5 nm; PMT voltage 400 V; scanning speed 240 nm/min. All protein samples were measured three times in parallel.

#### Particle size distribution of the Lys-CP mixture

The particle size distribution of the Lys-CP mixture was measured using a laser particle size analyzer (Ineas Physical Optics Instrument, Co. Ltd., WJ-60, Shanghai, China). Lys-CP solution was homogeneously mixed, poured into a stirred tank containing deionized water, and measured at a shading rate 1.50. All samples treated in triplicate.

### Effect of Lys on high concentration CP solutions

#### Determination of the dynamic rheology of Lys-CP

Frequency scan tests were carried out using a rheometer (HAAKE MARS40, Thermo Fisher Scientific, USA). Plates with a diameter of 35 mm and a rotor model P35/Ti-02180953 for use. The sample with a thickness of approximately 1 mm was placed on the plate. Set the temperature to 25 °C, the frequency range to 0.1–10 Hz, and the strain setting to 1%. The G′ and G″ values of the gel samples were measured and all samples tested were within the linear viscoelastic region. All samples were processed in triplicate.

#### SEM

Heat treatment Lys-CP samples were freeze-dried (LGJ-10, Songwon Huaxing Technology Development Co., Ltd., China) for 48 h. The dried samples were gold sprayed and observed under a field emission scanning electron microscope (ZEISS sigma 300, Germany) operating at 15 kV and a magnification of 5 kV.

### Statistical analysis

The protein content of CP, solubility, turbidity was calculated from the three determinants and expressed as mean ± standard deviation. Analyses were performed using SPSS (version 22.0, IBM SPSS Statistics, IBM Corporation, Armonk, NY, USA). After statistical analysis of variance (ANOVA) was used, the results were statistically interpreted at a significance of *P* < 0.05. Finally, graphs were plotted using Origin 2019 and Adobe Illustrator 2022.

## Conclusions

In this work, the effects of temperature and Lys on different concentrations (1%, 5%, and 9%, w/v) of CP suspensions were investigated. The higher the heat treatment temperature, the less CP was required to form thermally induced gels of CP. For low concentrations of CP suspensions, the addition of Lys reduces turbidity, solubility increases, FI decreases, particle size decreases, and moderates the generation of thermal aggregates. For high concentrations of CP suspensions, Lys prevents the formation of thermally induced gels and increases the fluidity of the solution, demonstrated the inhibitory effect of Lys on the thermal aggregation of CP. In conclusion, the results of this study provide a theoretical basis for improving the stability of coconut milk products during autoclaving and a solution for improving the production and processing of CP beverages.
